# Supplementary material for: Legionella effector MavC targets the Ube2N~Ub conjugate for noncanonical ubiquitination
Source: Nat Commun. 2020 May 12;11:2365. doi: 10.1038/s41467-020-16211-x (PMC7217864; doi:10.1038/s41467-020-16211-x)
Supplement: Supplementary file 1 — Supplementary Information [file 41467_2020_16211_MOESM1_ESM.pdf]

Supplementary Information

***Legionella* effector MavC targets the Ube2N~Ub conjugate for noncanonical ubiquitination**

Kedar Puvar, Shalini Iyer et al.

Supplementary Figure 1

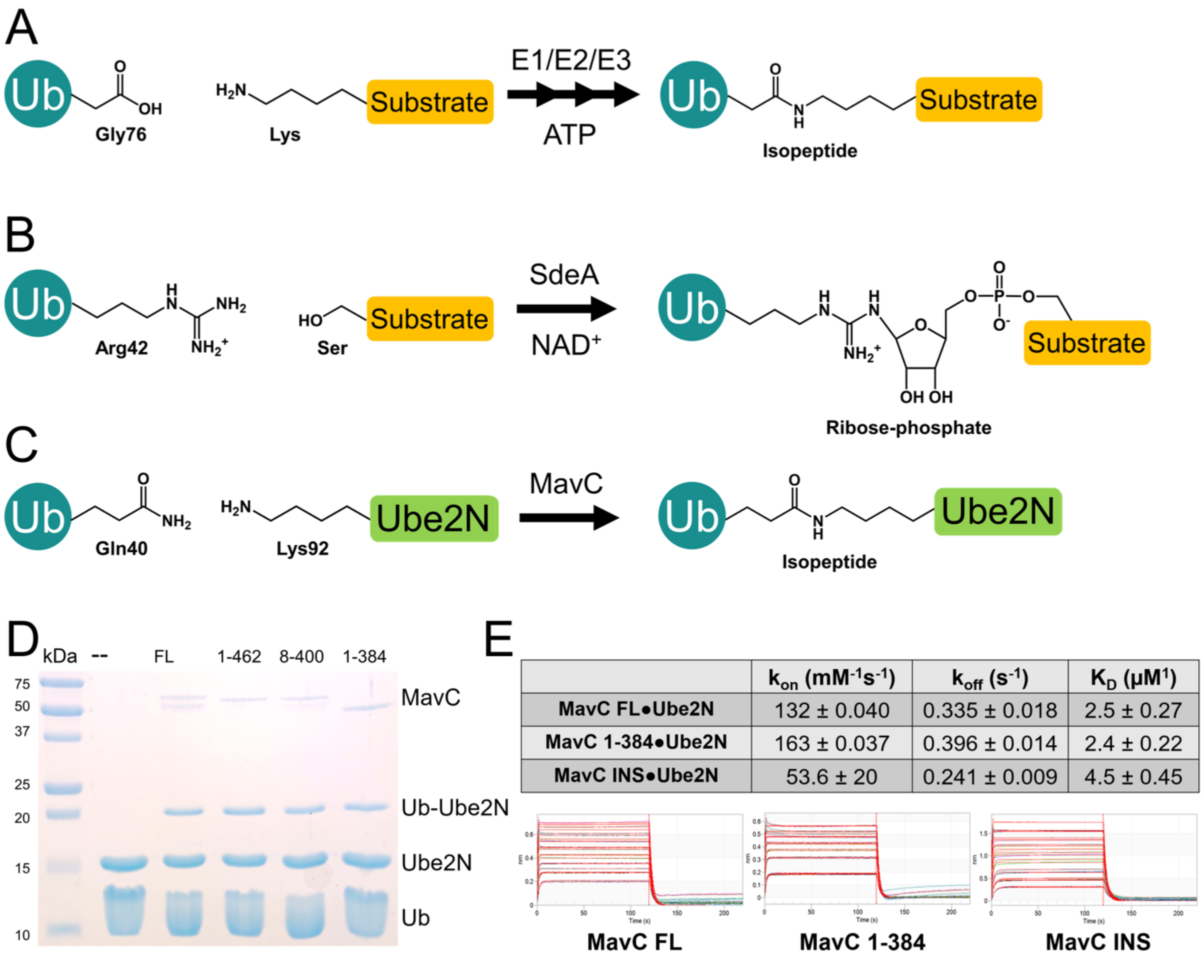

	k_{on} ($mM^{-1}s^{-1}$)	k_{off} (s^{-1})	K_D (μM^{1})
**MavC FL•Ube2N**	132 ± 0.040	0.335 ± 0.018	2.5 ± 0.27
**MavC 1-384•Ube2N**	163 ± 0.037	0.396 ± 0.014	2.4 ± 0.22
**MavC INS•Ube2N**	53.6 ± 20	0.241 ± 0.009	4.5 ± 0.45

Mechanisms of ubiquitination. A, General scheme of canonical ubiquitination, SidE-catalyzed ubiquitination (**B**), and MavC-catalyzed ubiquitination (**C**). **D**, Comparison of ubiquitinating ability of MavC constructs. Samples were analyzed by SDS-PAGE and visualized by Coomassie Blue staining. **E**, $MavC_{1\text{-}384}$ used for crystallization binds Ube2N similarly to the full-length construct. BLI curves and compiled on and off rates along with dissociation constants are provided.

Supplementary Figure 2

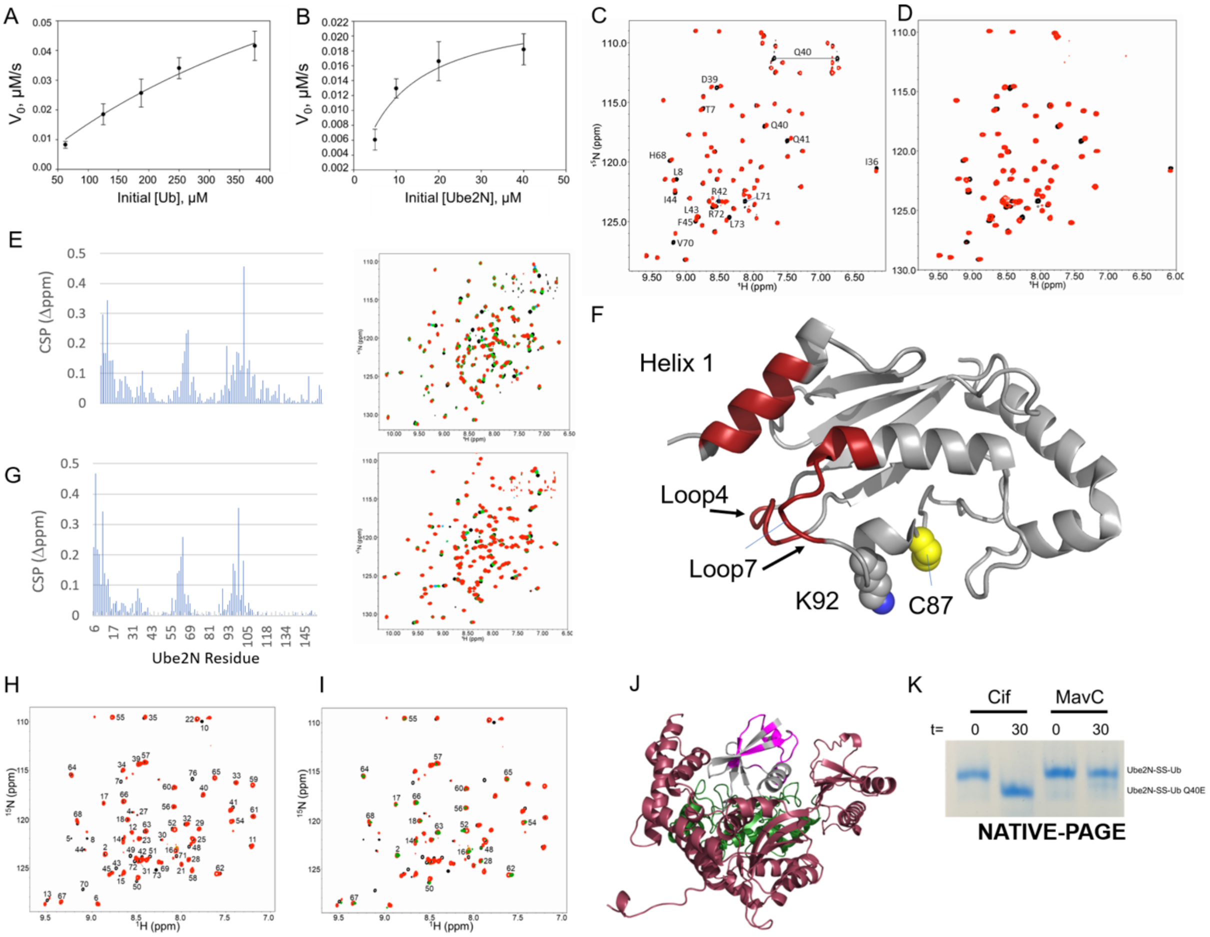

Analysis of free Ub and Ube2N binding to MavC. A, Michaelis-Menten curve comparing initial velocity of deamidation reaction vs initial [Ub]. n=3 independent *in vitro* experiments were carried out. Points indicate mean value and error bars indicate standard deviation. **B**, Michaelis-Menten curve comparing initial velocity of ubiquitination reaction vs initial [Ube2N]. n=3 independent *in vitro* experiments were carried out. Points indicate mean value and error bars indicate standard deviation. **C**, NMR spectral changes that occur upon conversion of Ub_{WT} to Ub_{Q40E}. The ^{1}H,^{15}N-HSQC spectrum of 150 □M ^{15}N-labeled Ub_{WT} is shown as black resonances. Full length $MavC_{WT}$ was added and incubated at room temperature for 1 hour and a second spectrum (red spectrum) was collected. The spectral overlay shows the chemical shift perturbation that occur upon conversion of substrate to product. The $Gln40^{Ub}$ side

chain resonances disappear and backbone resonances of residues that surround it are perturbed. **D**, 1H-15N TROSY spectral overlay of Ub_{WT} (black) and after incubation with catalytic amounts of MavC as in **C**. In this case, Ub subunits were separated from MavC by size-exclusion chromatography prior to data acquisition. The spectral changes are the same as that observed in panel (**C**). Therefore, all observed chemical shift perturbation can be explained by the conversion of substrate to product and cannot be ascribed to protein-protein interactions. **E**, Results of NMR titration of 2H,15N-Ube2N with C74A-$MavC_{1-384}$. Numerous chemical shift perturbations are observed in Ube2N upon addition of MavC. The magnitude of CSPs were determined for each residue as described in Methods. Corresponding spectra for the titration are shown in the right panel. Spectra were collected at 600 MHz with 150 mM ^{2}H,^{15}N-Ube2N + 0, 0.5, 1.0, and 1.5 equivalents of MavC. **F**, CSPs greater than one standard deviation from the mean (shown in E) are highlighted in red on a structure of Ube2N (PDB id 1j7d). The Ube2N residues most affected by addition of $MavC_{1-384}$ are located in Helix 1, Loop4, and Loop 7 and define a contiguous surface on the E2 that is recognized by MavC. This is the same surface recognized by certain eukaryotic E3 Ub-ligases that bind Ube2N. **G**, Results of NMR titrations of ^{1}H,^{15}N-Ube2N with unlabeled MavC insertion domain. Spectra were collected at 500 MHz with 150 mM ^{1}H,^{15}N-Ube2N + 0, 0.25, 0.5, and 1.0 equivalents of MavC. Comparison of (E) and (G) shown that the same regions of Ube2N are affected in both experiments. **H,** Spectral overlay ^{1}H-^{15}N-TROSY spectra of unbound wild-type ^{2}H,^{15}N-Ub (black spectrum) and ^{2}H,^{15}N-Ub-SS-Ube2N (red spectrum). Backbone resonance assignments are shown for wild-type Ub. The Ube2N subunit is not observed in these experiments as it is not labeled. In the Ub-SS-Ube2N conjugate a number of peaks are observed to shift or are lost due to exchange broadening. This occurs as the Ub subunit is in dynamic equilibrium between open conformations in which Ub is in an extended conformation and makes limited contact with Ube2N subunit, and closed conformations where Ub makes extensive contacts with the Ube2N subunit.[31] Resonances outside the contact region are largely unaffected and can be assigned by inspection. **I,** Spectrum of ^{2}H,^{15}N-Ub-SS-Ube2N/MavC complex (green spectrum) added to the overlay shown in (**A**). A general loss of peak intensity is observed across the spectrum due to the size of the complex (65kDa). In addition, a large number of peaks disappear from the spectrum as a result of exchange broadening. This behavior indicates that in solution, the Ub subunit in the Ub-SS-Ube2N/MavC complex is in dynamic equilibrium between

contact with MavC and other states. Thus, the Ub subunit is not rigidly docked in the complex. **J,** Ub resonances that remain in the spectrum of the of ^{2}H,^{15}N-Ub-SS-Ube2N/MavC complex overlay well with those of free Ub and can be assigned by inspection. Resonances not affected by complex formation are not likely to make contact with MavC. When plotted (in magenta) onto the Ub subunit (gray) in the context of Ub-SS-Ube2N/MavC complex, the unaffected resonances are largely solvent exposed and make little or no contact with MavC. MavC is shown in red, Ube2N in green. **K**, MavC does not appreciably deamidate Ub-SS-Ube2N. The disulfide conjugate was incubated with the indicated enzymes and reactions were analyzed by native-PAGE and visualized by Coomassie Blue. The known Ub deamidase Cif was included as a control.

Supplementary Figure 3

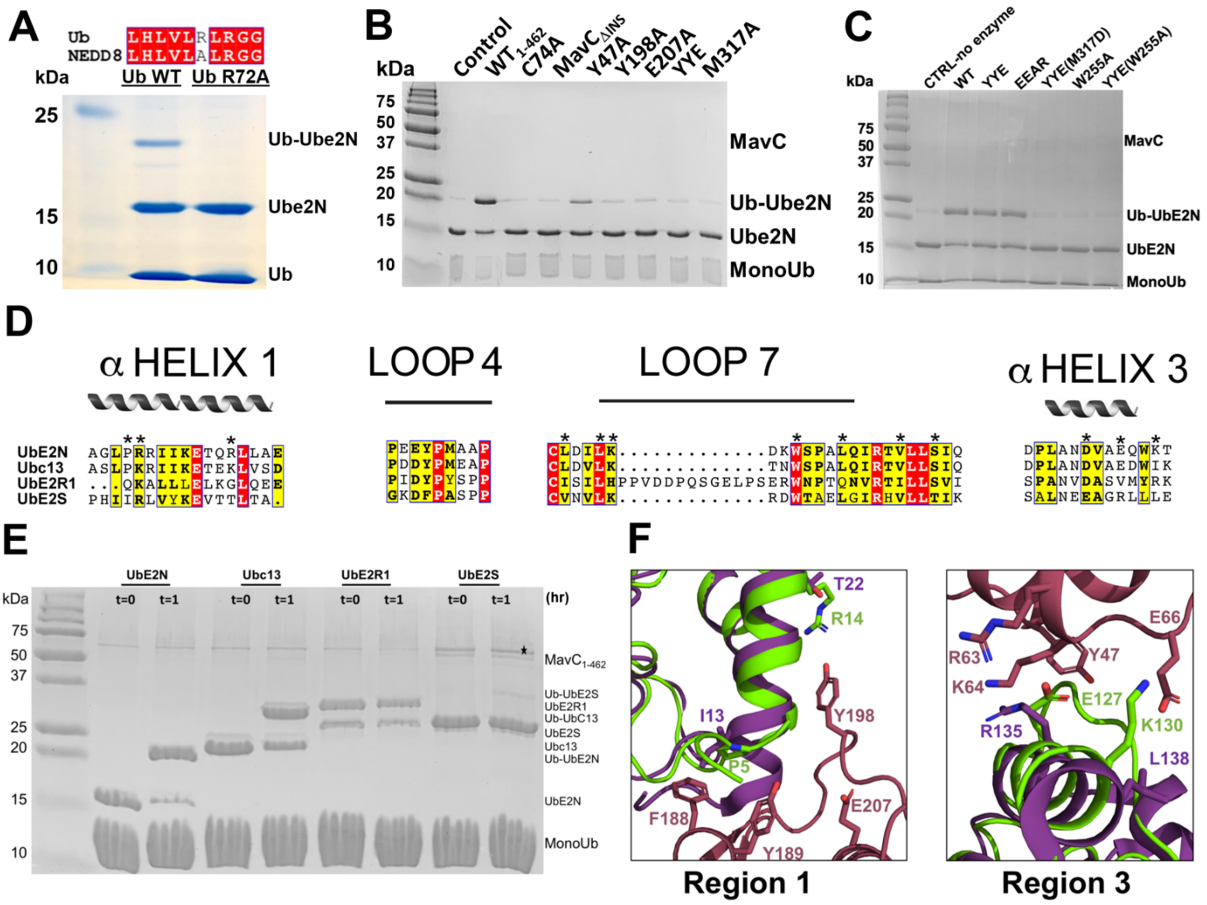

Analysis of MavC substrate selection. A, Importance of Arg72 in MavC recognition of Ub. Comparison of Ub WT and Ub R72A as a transglutamination substrate, with reactions analyzed by SDS-PAGE and visualized by Coomassie staining. Sequence alignment of Ub and NEDD8 is included. Conserved residues are highlighted in red. **B**, Mutational analysis of Ube2N binding residues. The YYE triple mutant was generated for use in biological experiments. Reactions were analyzed by SDS-PAGE and visualized with Coomassie Blue. **C**, Mutational analysis of YYE-based MavC mutants that affect Ube2N and Ub binding. Reactions were analyzed by SDS-PAGE and visualized with Coomassie Blue. **D**, Structure-based multiple sequence alignment of representative E2 enzymes: Ube2N, Ubc13 (yeast analog of Ube2N), UbE2R1

(conserved structure, lacks target Lys residue), UbE2S (conserved structure and has a target Lys residue) at key MavC-interacting regions. Similar residues are shown in black bold characters and have been boxed in yellow. Non-identical residues are in regular font. Identical residues are represented in white bold characters in a red box. Residues marked with an asterisk (*) represent the Ube2N residues that interact with MavC in our structure. Sequences were aligned using Clustal Omega[1] software and the alignment was rendered using ESPript 3.0 (http://espript.ibcp.fr/ESPript/ESPript/)[2]
E, Comparison of the ubiquitinating activity of wild-type MavC against the 4 chosen E2 enzymes. Reactions performed at two time points (0 and 1 hr) for each E2 enzyme were subjected to SDS-PAGE gel electrophoresis and visualized with Coomassie Blue.
F, Alignment of UbE2S with Ube2N in the MavC-Ub-Ube2N structure. MavC is depicted in burgundy, UbE2S in purple, and Ube2N in light green. Variable regions between Ube2N and UbE2S are shown, with contrasting residues between the two sequences depicted as stick models.

Supplementary Figure 4

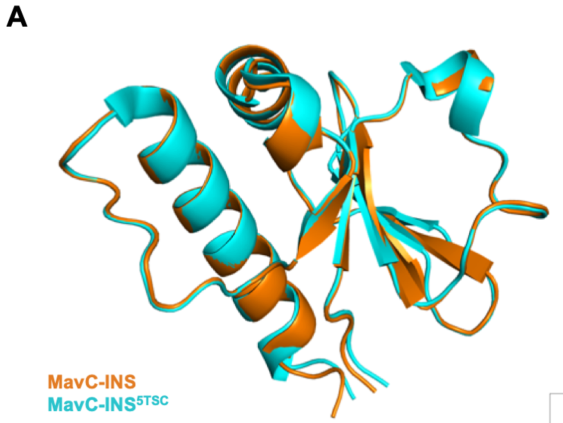

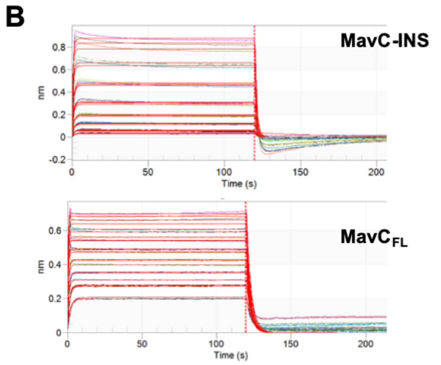

	k_{on} ($mM^{-1}s^{-1}$)	k_{off} (s^{-1})	K_D (μM)
MavC-INS - Ube2N	53.6 ± 20	0.241 ± 0.009	4.5 ± 0.45
$MavC_{FL}$ - Ube2N	132 ± 67	0.331 ± 0.018	2.5 ± 0.27
MavC-ΔINS - Ube2N	ND		ND

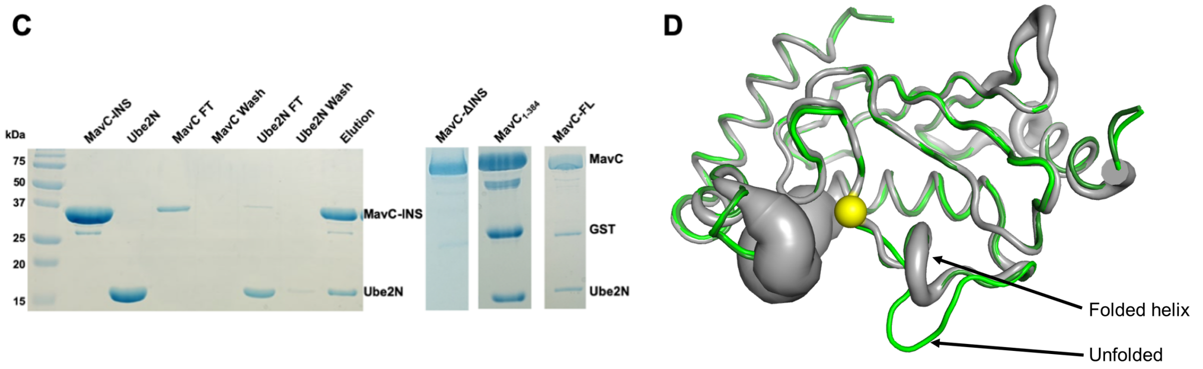

The MavC insertion domain is an independent Ube2N-binding motif. A, Alignment of the structure of the MavC insertion domain (PDB code: 6P5H; orange) with that from the full-length protein (PDB code: 5TSC; cyan). **B**, Biolayer interferometry association and dissociation curves of $MavC_{INS}$ (mounted on the biosensor) with UbE2N, and table containing the calculated binding parameters. **C,** Pulldown experiments of GST-tagged MavC constructs with UbE2N. Samples were run on SDS-PAGE gels and visualized with Coomassie Blue. **D**, Sausage plot depicting variable regions of Ube2N (diameter of gray cartoon corresponds to maximum rmsd of the calculated average structure of Ube2N) aligned with the structure of Ube2N moiety in the product-bound complex of MavC, given in green.

Supplementary Figure 5

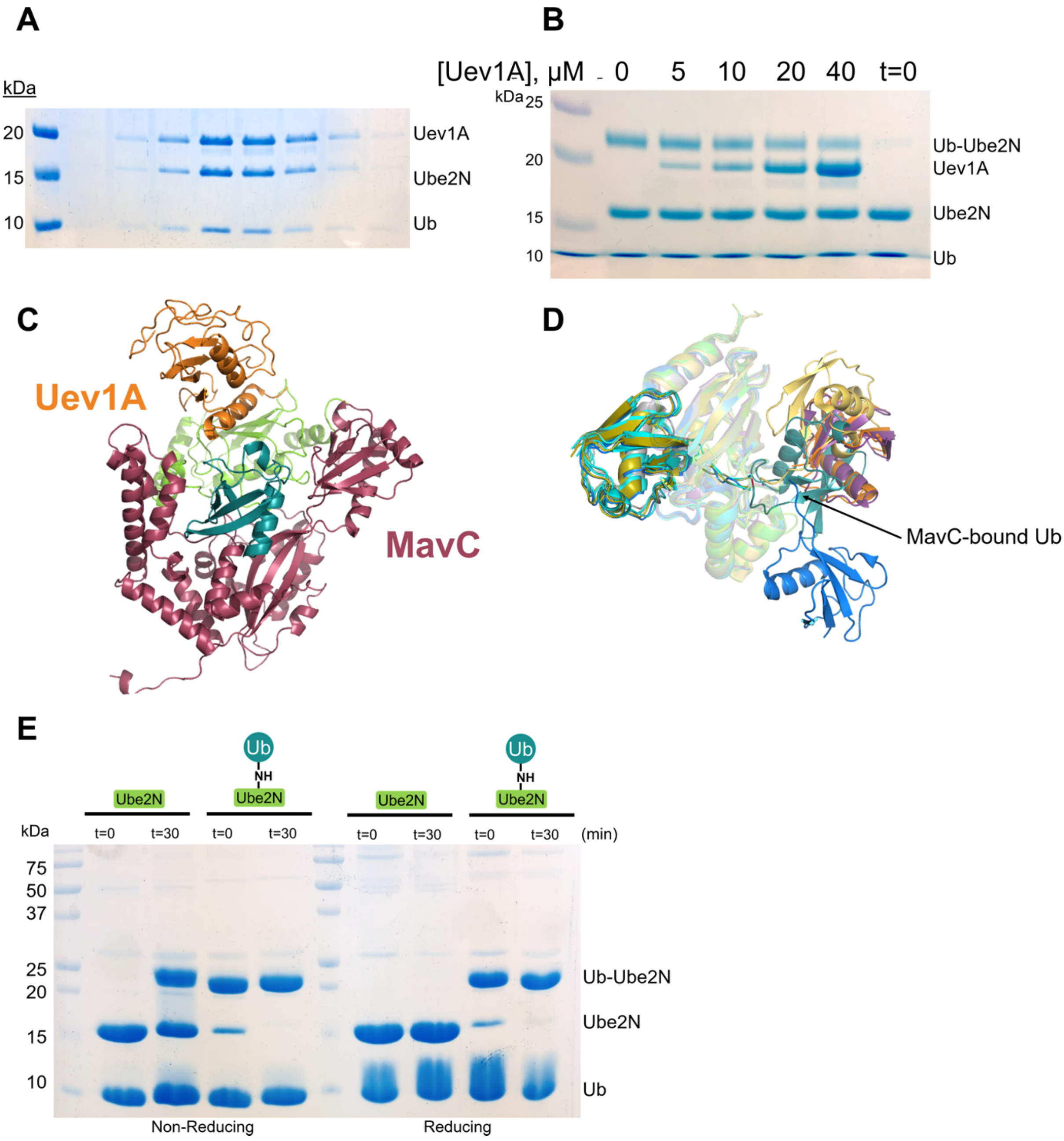

Analysis of Ub-Ube2N product and Uev1A binding. **A**, Co-elution of Uev1A with Ube2N-SS-Ub. Size-excluded fractions of the Uev1A/Ube2N-SS-Ub complex were analyzed by a reducing SDS-PAGE and visualized with Coomassie Blue. **B**, MavC

reaction with Ube2N-SS-Ub in the presence of increasing amounts of the heterodimeric binding partner Uev1A. MavC was added after incubation of substrate with Uev1A. Reactions were quenched with SDS-PAGE loading buffer and visualized with Coomassie Blue. **C**, Ribbon representation of the MavC-Ube2N-SS-Ub complex with Uev1A (orange; PDB code 2GMI) docked onto it to show that the disulfide substrate mimic doesn't affect heterodimerization between Ube2N and Uev1A. **D**, Overlay of the MavC-Ube2N-SS-Ub complex with all the structures of Ub~Ube2N(Ubc13) from the PDB. The MavC-bound Ub (from our complex) is shown in teal. PDB codes for the structures used for the alignment are listed in **Supplementary Table 3**. **E**, Charging assay comparing the ability of Ube2N and the MavC product, Ub-Ube2N to become charged by the E1 enzyme. Reactions were quenched with either non-reducing (left) or reducing (right) SDS-PAGE loading buffer at the indicated time points. Gels were visualized by Coomassie Blue.

Supplementary References

1. Sievers, F. *et al.* Fast, scalable generation of high-quality protein multiple sequence alignments using Clustal Omega. *Mol. Syst. Biol.* **7**, 539 (2011).

2. Robert, X. and Gouet, P. Deciphering key features in protein structures with the new ENDscript server. *Nucl. Acids Res*. **42**, W320-W324 (2014).

Supplementary Table 1: Primer sequences for all the constructs used in the study.

#	primer name	primer sequence (5' ➔ 3')
	**MavC FL (1-482)**	
**1**	FWD	CGGGATCCATGACAACTTCCAAGCTTG
**2**	REV	CGGTCGACTCACTTATCACGAAGAAC
	**MavC 1-462**	
**3**	FWD	GCTATGCTGGATCCATGACAACTTCCAAGCTT
**4**	REV	GCTATGCTCTCGAGTTAAGCCTTGATTTCTGCCAG
	**MavC 1-384**	
**5**	FWD	GCTATGCTGGATCCATGACAACTTCCAAGCTT
**6**	REV	GCTATGCTCTCGAGTTACTCTATTAGAAGTCGCAT
	**FLAG-MavC**	
**7**	FWD	CGGGATCCATGACAACTTCCAAGCTTG
**8**	REV	CGGTCGACTCACTTATCACGAAGAAC
	**FLAG-TRAF6**	
**9**	FWD	CGAGATCTATGAGTCTGCTAAACTGTG
**10**	REV	CGGTCGACCTATACCCCTGCATCAGTA
	**MavC 1-482 - 12xHIS**	
**11**	FWD	GAGATATACCATGGGCAGCAGCATGACAACTTCCAAGCTTGAAAAAACG

**12**	REV1	ATGATGATGATGATGATGCTTATCACGAAGAACTAACCCGTATTG
**13**	REV2	ATGATGATGATGATGATGATGATGATGATGATGCTTATCACGAAG
**14**	REV3	CAGCCGGATCCTCGAGTCAATGATGATGATGATGATGATGATGATGATG
	**MavC INS - 12xHIS**	
**15**	FWD	GAGATATACCATGGGCAGCAGCATGGATCGATTTAATGCTCCTCAAAAATACCAA
**16**	REV	ATGATGATGATGATGATGATGATGATGATGATGATGCCAACAATCTATGTTTTTCATTTTAGCTTTAACTATCTCAC
	**MavC DINS- 12xHIS**	
**17**	REV1	AAAAGAATGGGTTGCTGTATCCATTTCAACCCTGACCAGTAAATTTTT
**18**	FWD2	CTGGTCAGGGTTGAAATGGATACAGCAACCCATTCTTTTACTATATTTGTTCC
	**MavC 1-384 - 12xHIS**	
**19**	FWD	GAGATATACCATGGGCAGCAGCATGACAACTTCCAAGCTTGAAAAAACG
**20**	REV	ATGATGATGATGATGATGATGATGATGATGCTCTATTAGAAGTCGCATCTCAAGTTGTTTAGTCA
	**MavC mutants**	
**21**	MavC_Y47A-FWD	GAGGCAGAAATTTCGGTTGGAGCTGTAAAAAAAATGGG
**22**	MavC_Y47A-REV	CCCATTTTTTTTACAGCTCCAACCGAAATTTCTGCCTC
**23**	MavC_V61-FWD	GAAAGAAAATATTGCTGAAGCAGTTCGAAAATCTGAAATG
**24**	MavC_V61A-REV	CATTTCAGATTTTCGAACTGCTTCAGCAATATTTTCTTTC
**25**	MavC_R63A-FWD	AAAATATTGCTGAAGTAGTTGCAAAATCTGAAATGACTCAACCCACAA
**26**	MavC_R63A-REV	TTGTGGGTTGAGTCATTTCAGATTTTGCAACTACTTCAGCAATATTTT

**27**	MavC_K64A-FWD	ATTGCTGAAGTAGTTCGAGCATCTGAAATGACTCAACCC
**28**	MavC_K64A-REV	GGGTTGAGTCATTTCAGATGCTCGAACTACTTCAGCAAT
**29**	MavC_E66A-FWD	GTAGTTCGAAAATCTGCAATGACTCAACCCACAAACAG
**30**	MavC_E66A-REV	CTGTTTGTGGGTTGAGTCATTGCAGATTTTCGAACTAC
**31**	MavC_N72A_FWD	GACTCAACCCACAGCCAGCTGCGGAAAAGCGAG
**32**	MavC_N72A-REV	CTCGCTTTTCCGCAGCTGGCTGTGGGTTGAGTC
**33**	MavC_S73A-FWD	CAACCCACAAACGCCTGCGGAAAAGCGAGCAAT
**34**	MavC_S73A_REV	ATTGCTCGCTTTTCCGCAGGCGTTTGTGGGTTG
**35**	MavC_N79A-FWD	AACAGCTGCGGAAAAGCGAGCGCTGAAGTGTGTGATTT
**36**	MavC_N79A-REV	AAATCACACACTTCAGCGCTCGCTTTTCCGCAGCTGTT
**37**	MavC_R121-FWD	CAATAAAAATTTACTGGTCGCGGTTGAAATGGATCGATTT
**38**	MavC_R121-REV	AAATCGATCCATTTCAACCGCGACCAGTAAATTTTTATTG
**39**	MavC_R126-FWD	GGTCAGGGTTGAAATGGATGCATTTAATGCTCCTCA
**40**	MavC_R126A-REV	TGAGGAGCATTAAATGCATCCATTTCAACCCTGACC
**41**	MavC_F188A-FWD	GCTATTACCACCCCTGCTTATCAAAATTATATTCTTTATGATGAG
**42**	MavC_F188A-REV	CTCATCATAAAGAATATAATTTTGATAAGCAGGGGTGGTAATAGC
**43**	MavC_Y189A-FWD	GCTATTACCACCCCTTTTGCTCAAAATTATATTCTTTATGATGAG
**44**	MavC_Y189A-REV	CTCATCATAAAGAATATAATTTTGAGCAAAAGGGGTGGTAATAGC
**45**	MavC_Y198A-FWD	TTATATTCTTTATGATGAGGCTATTGATCCGGAAGAAAGTGC

**46**	MavC_Y198A-REV	GCACTTTCTTCCGGATCAATAGCCTCATCATAAAGAATATAA
**47**	MavC_E207A-FWD	GGAAGAAAGTGCTGAGGCAGCGGCCATGTTTGAAA
**48**	MavC_E207A-REV	TTTCAAACATGGCCGCTGCCTCAGCACTTTCTTCC
**49**	MavC_T230A-FWD	CATAGATTGTTGGACAGCAGCCCATTCTTTTACTATA
**50**	MavC_T230A-REV	TATAGTAAAAGAATGGGCTGCTGTCCAACAATCTATG
**51**	MavC_Y254A-FWD	CATTATATCCTTACCAGGCAGCTTGGACCAGCCATACTTTGC
**52**	MavC_Y254A-REV	GCAAAGTATGGCTGGTCCAAGCTGCCTGGTAAGGATATAATG
**53**	MavC_W255A-FWD	GCACATTATATCCTTACCAGGCATATGCGACCAGCCATACTT
**54**	MavC_W255A-REV	AAGTATGGCTGGTCGCATATGCCTGGTAAGGATATAATGTGC
**55**	MavC_M317A-FWD	CATGTTTTTTGTACCGGGGCGAACGAAAAATTCTCGCC
**56**	MavC_M317A-REV	GGCGAGAATTTTTCGTTCGCCCCGGTACAAAAAACATG
**57**	MavC_M317D-FWD	GAACATGTTTTTTGTACCGGGGACAACGAAAAATTCTCGCCATTACG
**58**	MavC_M317D-REV	CGTAATGGCGAGAATTTTTCGTTGTCCCCGGTACAAAAAACATGTTC
	**Ub constructs**	
**59**	Ub-G76C-FWD	CTCCGTCTCAGAGGTTGCTAATAGAATTCG
**60**	Ub-G76C-REV	CGAATTCTATTAGCAACCTCTGAGACGGAG
**61**	Ub_R72A-FWD	GCACCTGGTGCTCGCTCTCAGAGGTGGG
**62**	Ub_R72A-REV	CCCACCTCTGAGAGCGAGCACCAGGTGC

	**MavC INS**	
**63**	FWD	GGATTCCCATAGGGATCCAATGCTCCTCAAAAATACCAA
**64**	REV	TTCAGGGCCTATCTCGAGTCAATCTATGTTTTTCATTTTAGC
	**Ube2N**	purchased from Addgene
	**Ubc13**	purchased from Addgene
	**Ube2S, Ube2R1, Uba1, Ub**	kind gift from Genentech Corporation (USA)
	**UeV1a**	kind gift from the Yasuke Sato (University of Tokyo, Japan)

Supplementary Table 2 Data collection, processing and refinement statistics

	$MavC_{1-384}$_Ub-SS-UbE2N early complex	$MavC_{1-384}$_Ub-SS-UbE2N attacking complex (I)	$MavC_{1-384}$_Ub-SS-UbE2N attacking complex (II)	$MavC_{1-384}$_Ub-UbE2N product complex	$MavC_{INS}$ domain
**PDB ID**	**6UMP**	**6ULH**	**6UMS**	**6P5B**	**6P5H**
**Resolution range**	30.75 - 2.8 (2.85 - 2.80)	25.34 - 1.968 (2.039 - 1.968)	38.63 - 2.34 (2.43 - 2.34)	30.36 -2.01 (2.17 - 2.01)	39.23 -1.53 (1.59 -1.53)
**Space group**	P 6_5	R 3:H	C222_1	P 6_5	P 3_1
**Unit cell**	a=b=150.478 Å c=53.357 Å γ=120°	a=b=171.86 Å c=58.36 Å γ=120°	a=98.55 Å b=104.37 Å c=124.44 Å α=β=γ=90°	a=b=147.84 Å c=53.23 Å γ=120°	a=b=52.03 Å c=79.75 Å γ=120°
**Total reflections**	1888913	1107581	694463	1383292	298548
**Unique reflections**	19287	45560	27260	39130	36418
**Multiplicity**	10.2 (9.7)	5.8 (5.5)	7.1 (5.3)	10.4 (10.0)	8.2
**Completeness (%)**	99.75 (99.88)	99.91 (99.74)	99.83 (97.83)	99.16 (97.89)	99.96 (100.00)
**Mean I/sigma(I)**	15.13 (1.17)	10.5 (1.7)	12.24 (1.45)	22.0 (2.5)	24.9(4.1)
**Wilson B-factor**	66.93	31.44	40.03	32.82	17.62
**R-merge**	0.201	0.098	0.187	0.136	0.054
**CC1/2 (last shell)**	0.514	0.475	0.433	0.777	0.932
**Reflections (refinement)**	17235 (1696)	45553 (4570)	27196 (2620)	38834 (3802)	36383 (3635)
**Reflections (R-free)**	1738 (174)	2007 (194)	1286 (127)	2020 (177)	1995 (194)
**R-work**	0.2426 (0.3433)	0.1767 (0.2483)	0.2065 (0.3013)	0.1782 (0.2235)	0.2001 (0.2461)
**R-free**	0.3013 (0.3830)	0.2297 (0.3211)	0.2437 (0.3631)	0.2316 (0.3093)	0.1943 (0.2637)
macromolecules	4608	4655	4520	4784	1620
solvent	0	298	168	300	361
**RMS bonds (Å)/ angles (°)**	0.003 / 0.59	0.007 / 0.86	0.002 / 0.49	0.008 / 0.89	0.007 / 1.17
**Ramachandran - favored**	95.31	98.81	97.79	97.87	98.96
**Ramachandran - allowed**	4.69	1.19	2.21	2.13	1.04
**Average B-factor ($Å^2$)**	67.39	40.96	59.17	39.7	23.12

Supplementary Table 3: Ube2N~Ub structures in PDB used in this analysis

Code	Title
2GMI	Mms2/Ubc13~Ubiquitin
5VNZ	Structure of a TRAF6-Ubc13~Ub complex
5VO0	Structure of a TRAF6-Ubc13~Ub complex
4DHJ	The structure of a ceOTUB1 ubiquitin aldehyde UBC13~Ub complex
4DHZ	The structure of h/ceOTUB1-ubiquitin aldehyde-UBC13~Ub
5EYA	TRIM25 RING domain in complex with Ubc13-Ub conjugate
5AIT	A complex of of RNF4-RING domain, UbeV2, Ubc13-Ub (isopeptide crosslink)
5AIU	A complex of RNF4-RING domain, Ubc13-Ub (isopeptide crosslink)
4WHV	E3 ubiquitin-protein ligase RNF8 in complex with Ubiquitin-conjugating enzyme E2 N and Polyubiquitin-B
6S53	Crystal structure of TRIM21 RING domain in complex with an isopeptide-linked Ube2N~ubiquitin conjugate
