# Supplementary material for: Legionella effector MavC targets the Ube2N~Ub conjugate for noncanonical ubiquitination
Source: Nat Commun. 2020 May 12;11:2365. doi: 10.1038/s41467-020-16211-x (PMC7217864; doi:10.1038/s41467-020-16211-x)

Original/Uncropped Gels

***Legionella* effector MavC targets the Ub~Ube2N conjugate for noncanonical ubiquitination**

Kedar Puvar [1*], Shalini Iyer [1*], Sebastian Kenny[1], Kristos I. Negrón Terón[1], Jiaqi Fu[2], Zhao-Qing Luo [2], Peter Brzovic[3], Rachel Klevit[3¶], Chittaranjan Das [1¶]

[1]Department of Chemistry, Purdue University, West Lafayette, IN 47907 USA

[2]Purdue Institute for Inflammation, Immunology and Infectious Disease and Department of Biological Sciences, Purdue University, West Lafayette, IN 47907, USA

[3]Department of Biochemistry, University of Washington, Seattle, Washington 98195, USA

*These authors contributed equally to this work

[¶]Correspondence: cdas@purdue.edu

klevit@uw.edu

Figure 2E

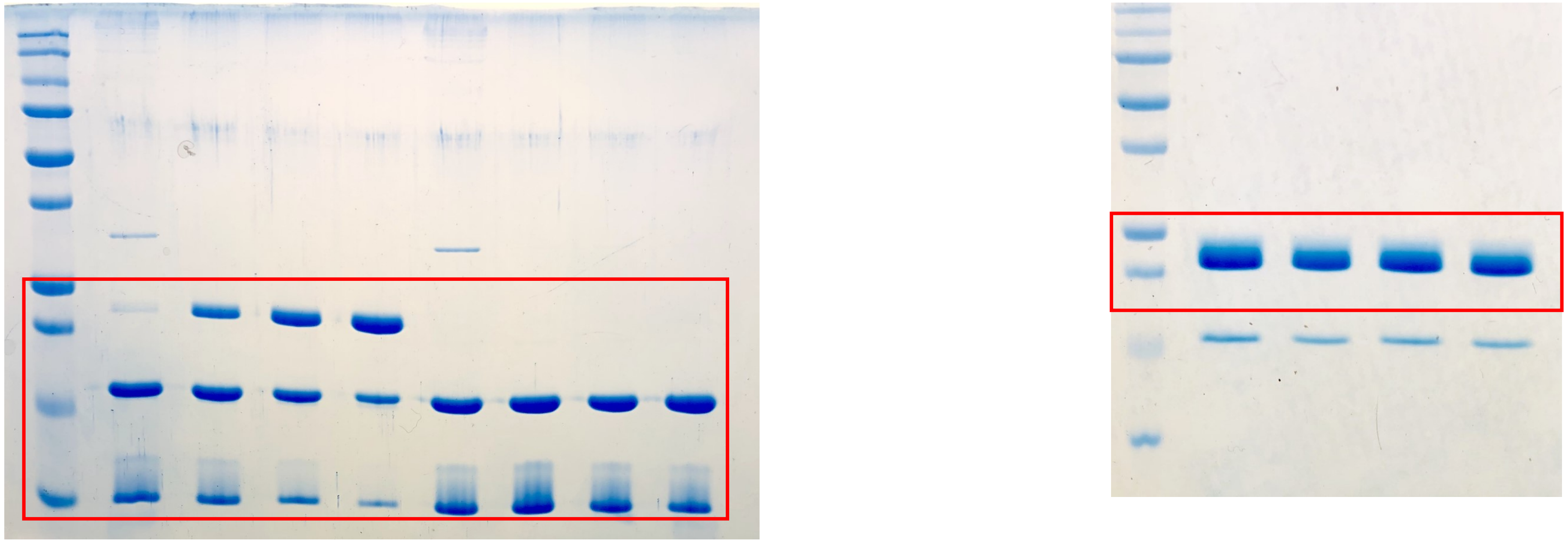

Figure 4C

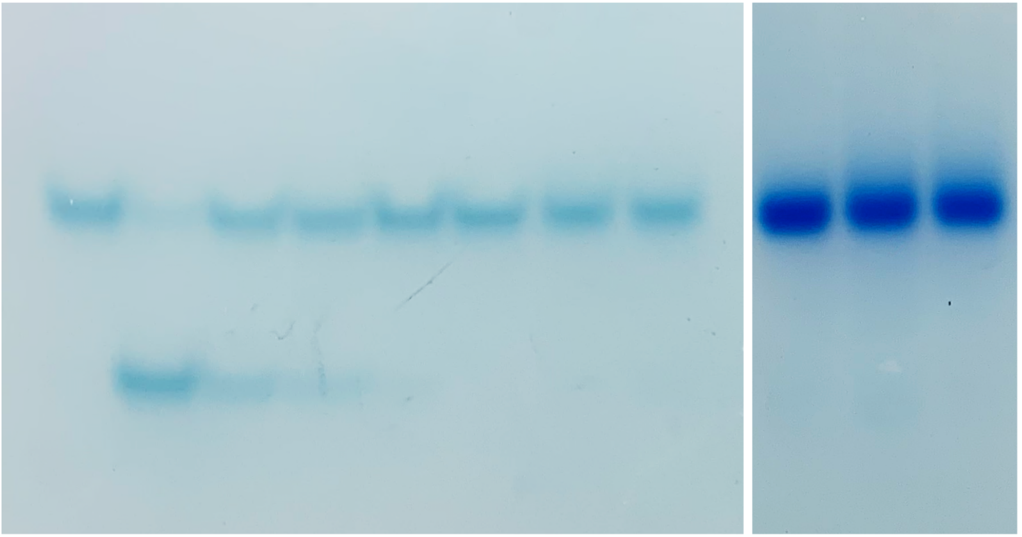

Figure 4D

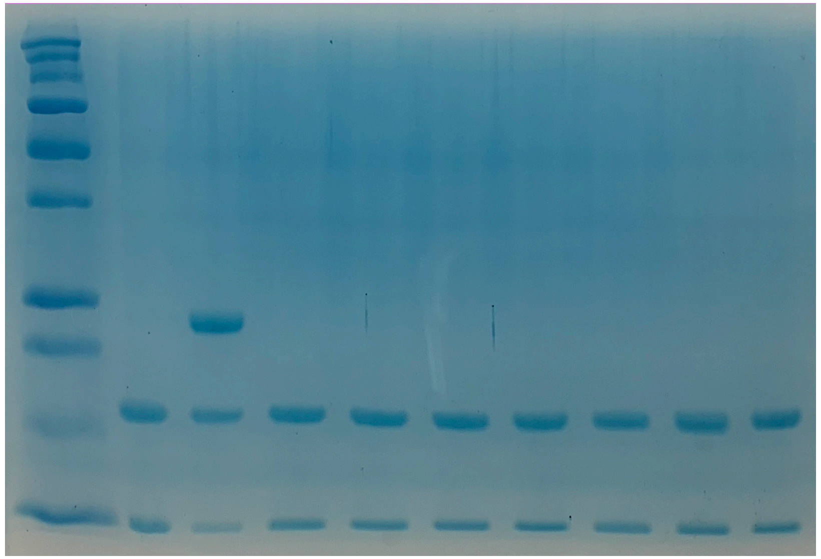

Figure 5D

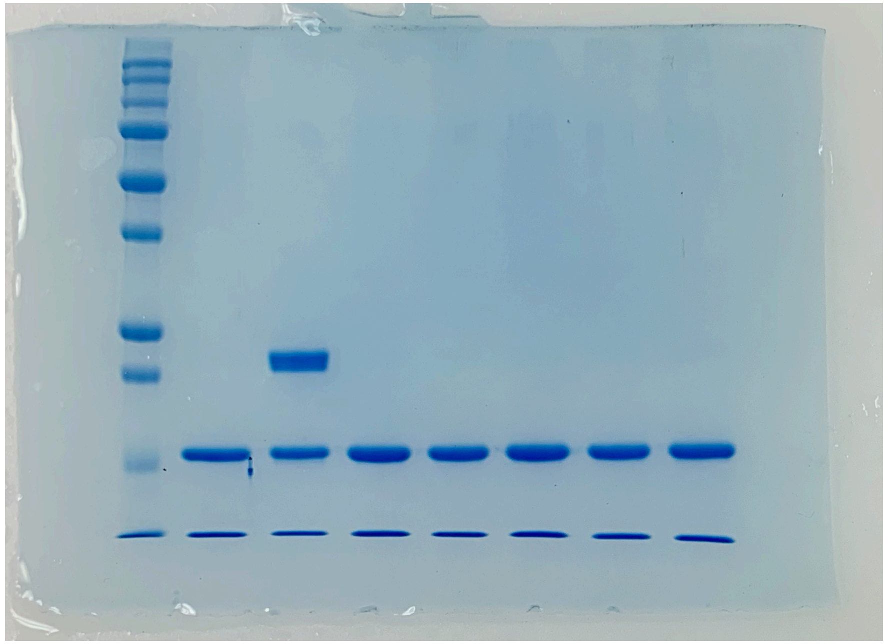

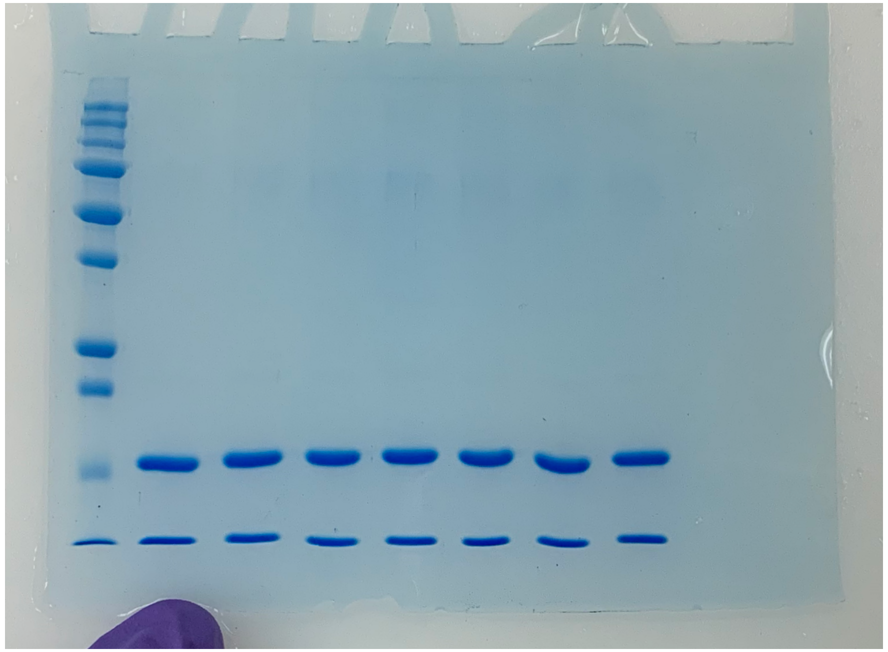

Figure 5E

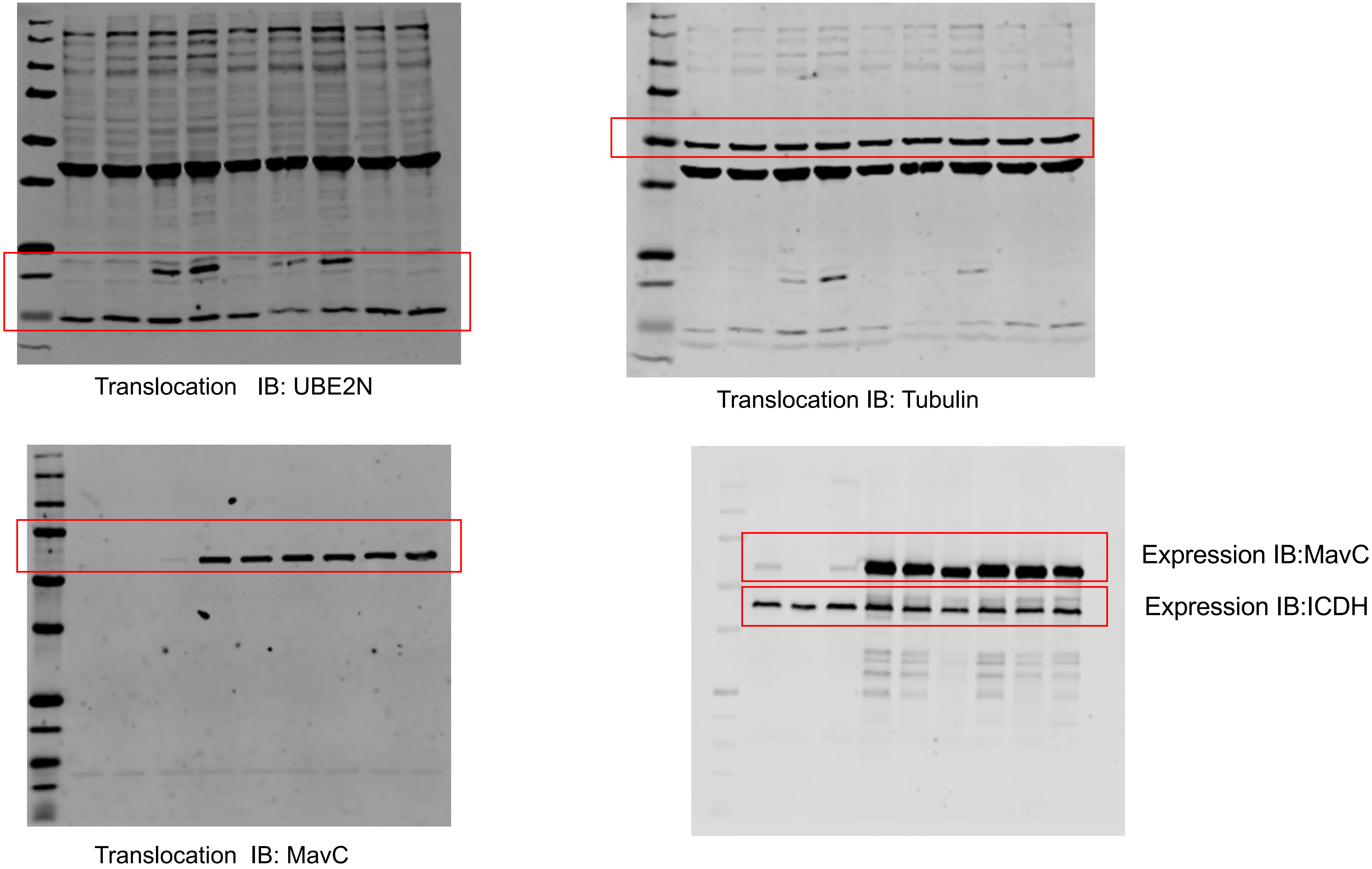

Translocation IB: UBE2N
Translocation IB: Tubulin
Translocation IB: MavC
Expression IB:MavC
Expression IB:ICDH

Figure 5F

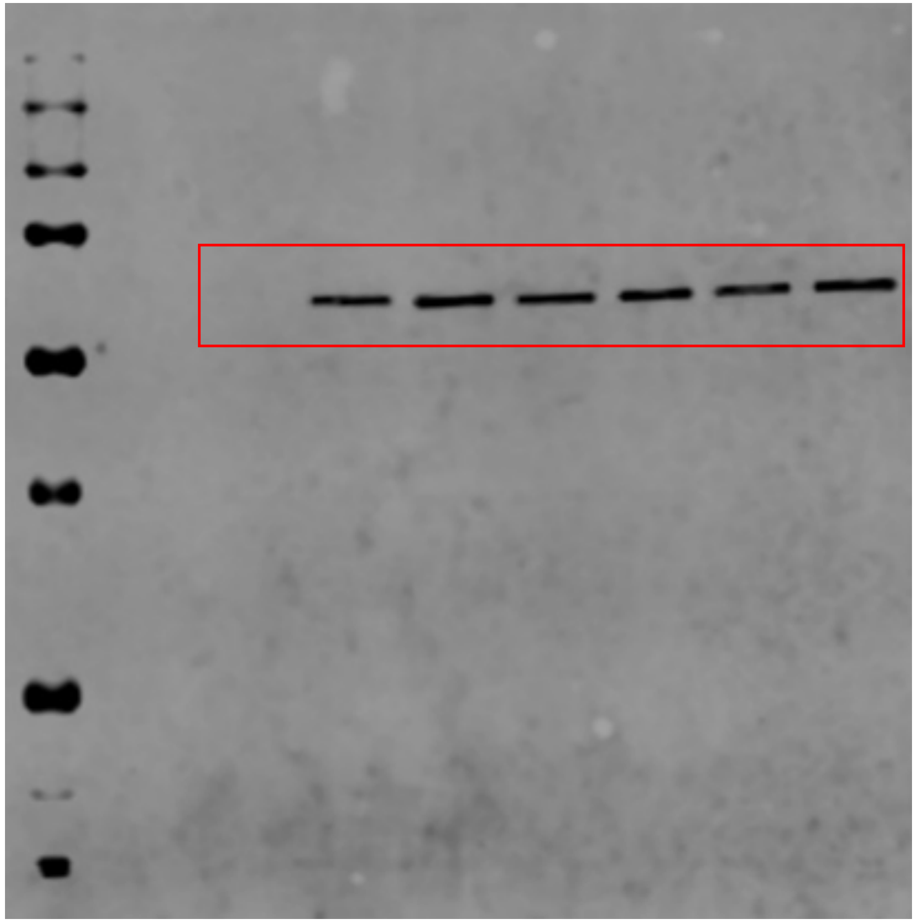

IB:MavC

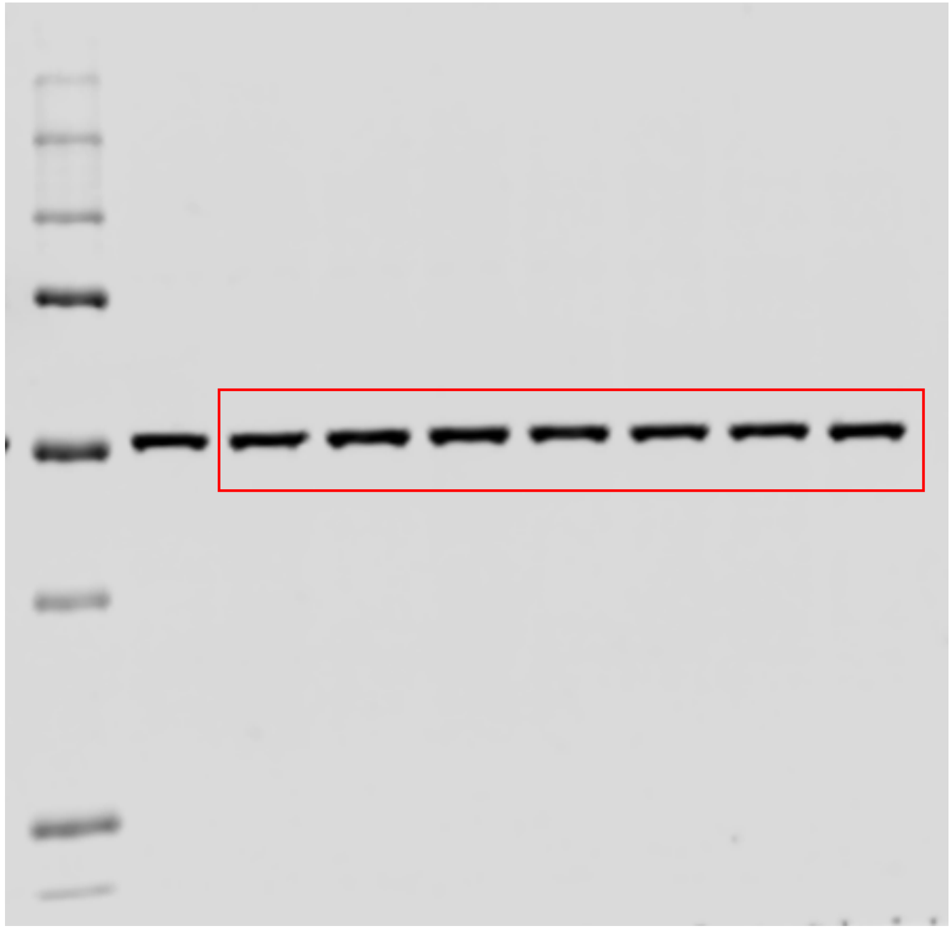

IB:Tubulin

Supplementary Figure 3A

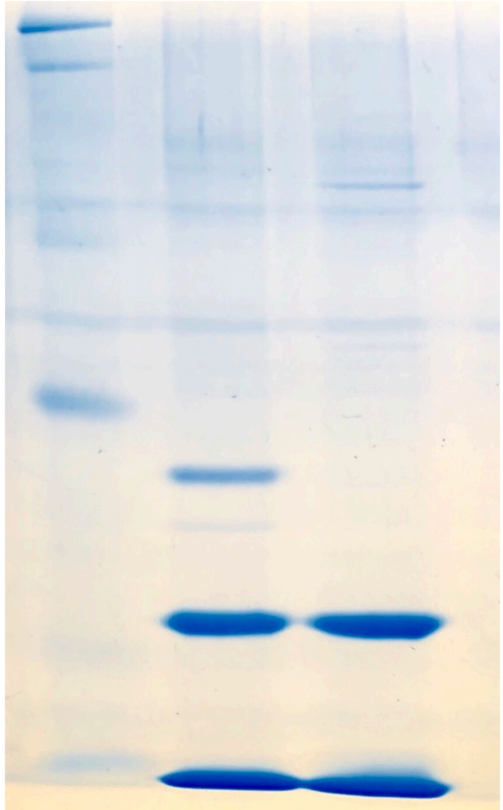

Supplementary Figure 3B

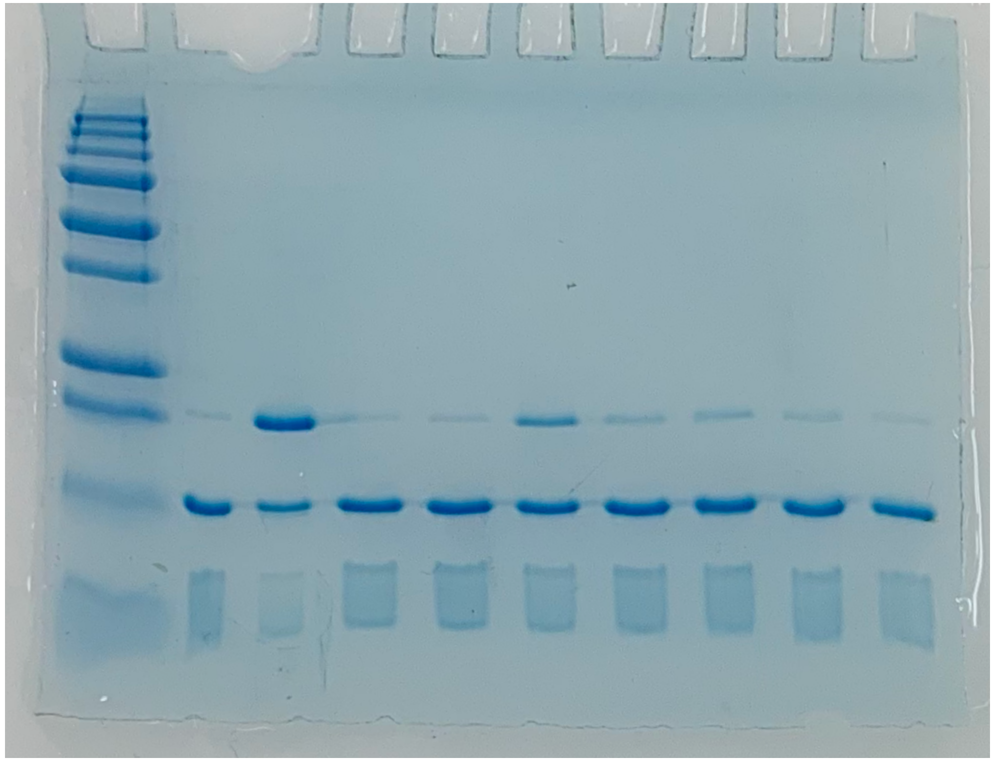

Supplementary Figure 3C

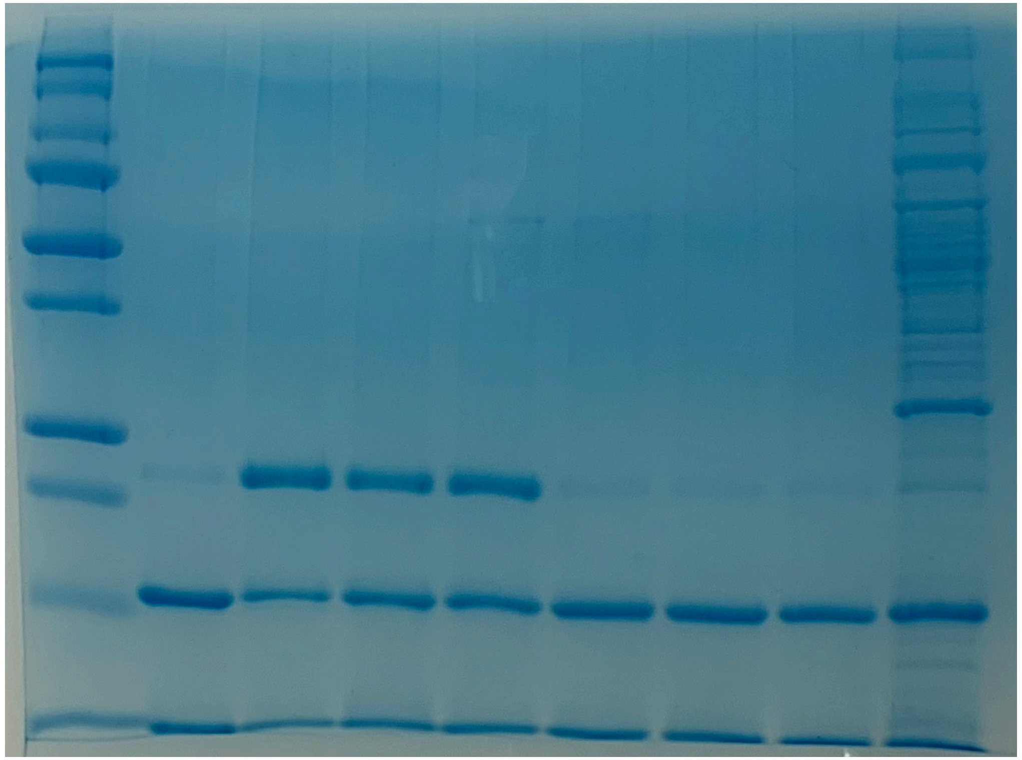

Supplementary Figure 3E

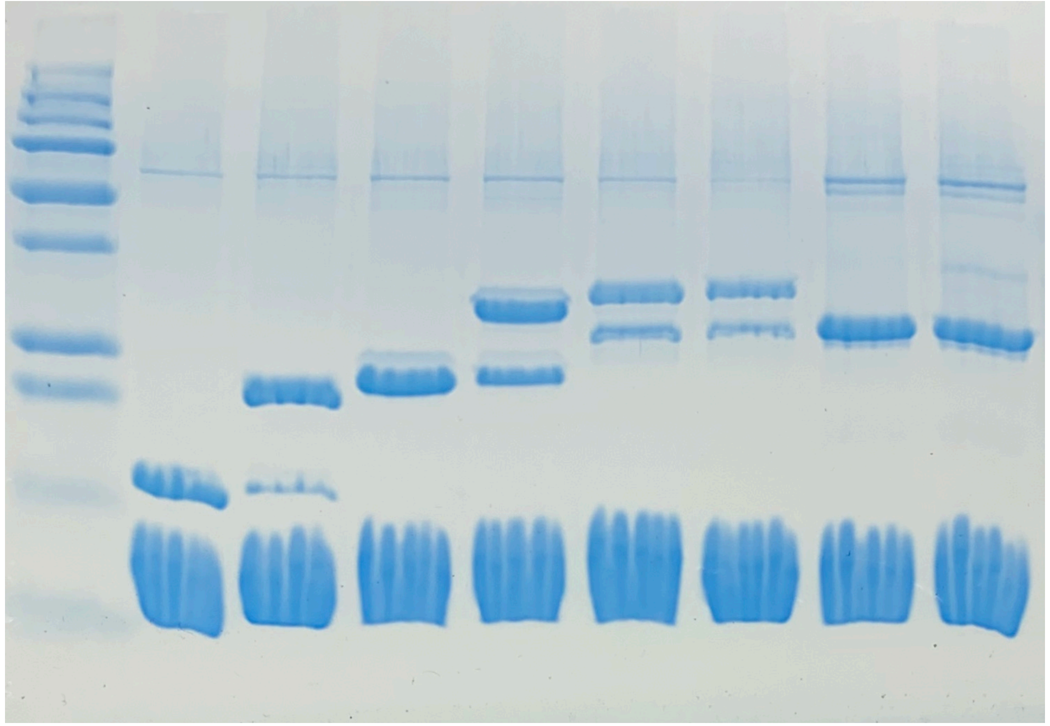

Supplementary Figure 4C

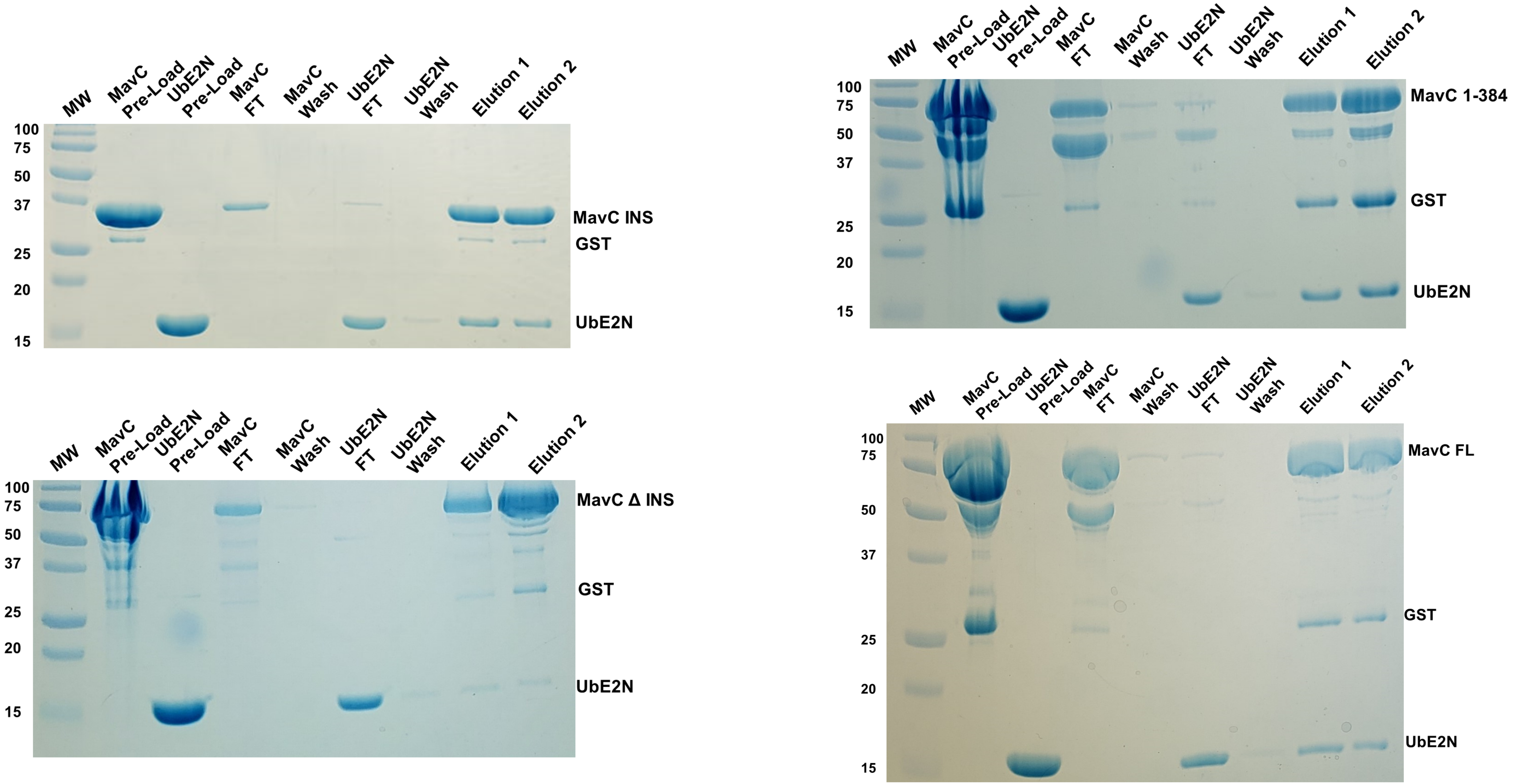

Supplementary Figure 5A

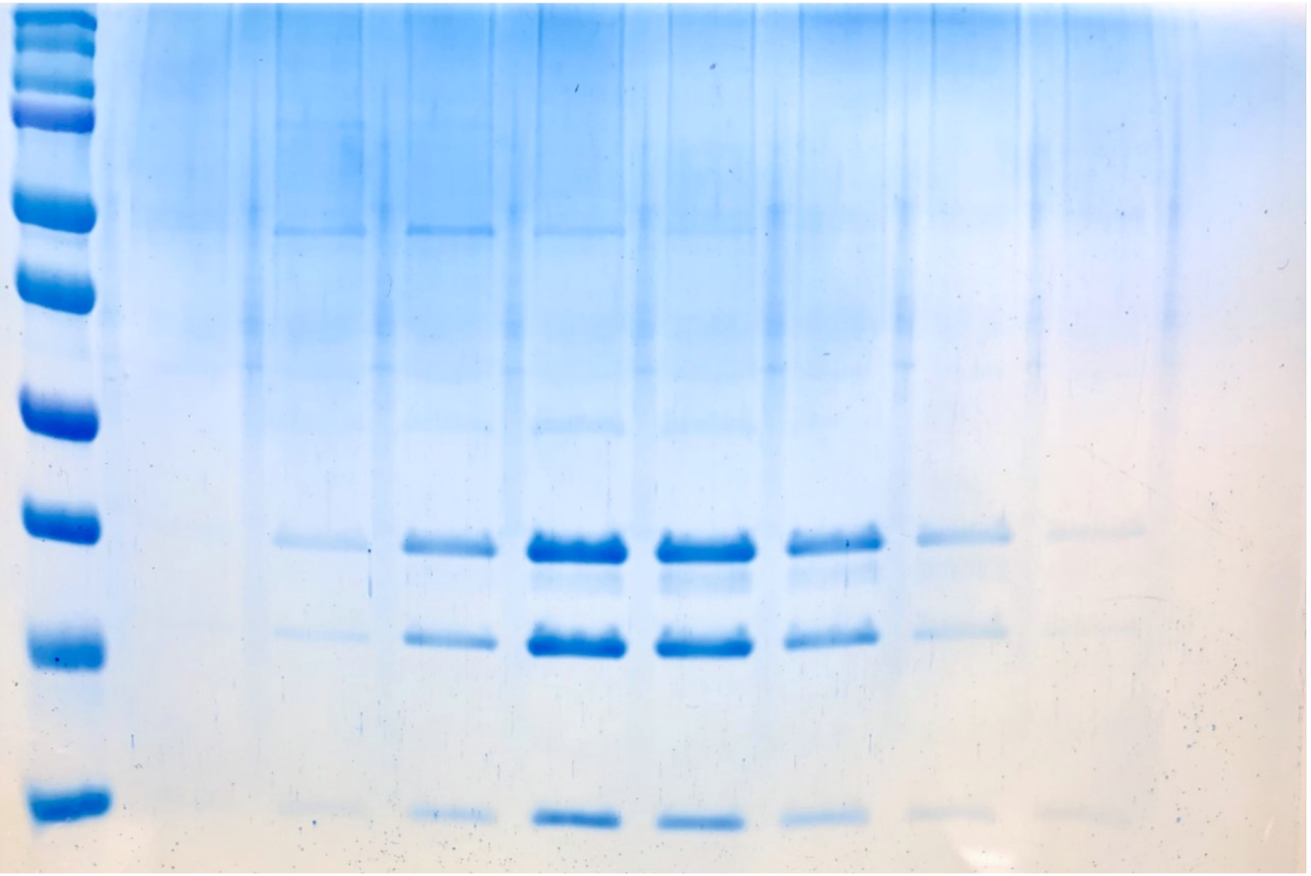

Supplementary Figure 5B

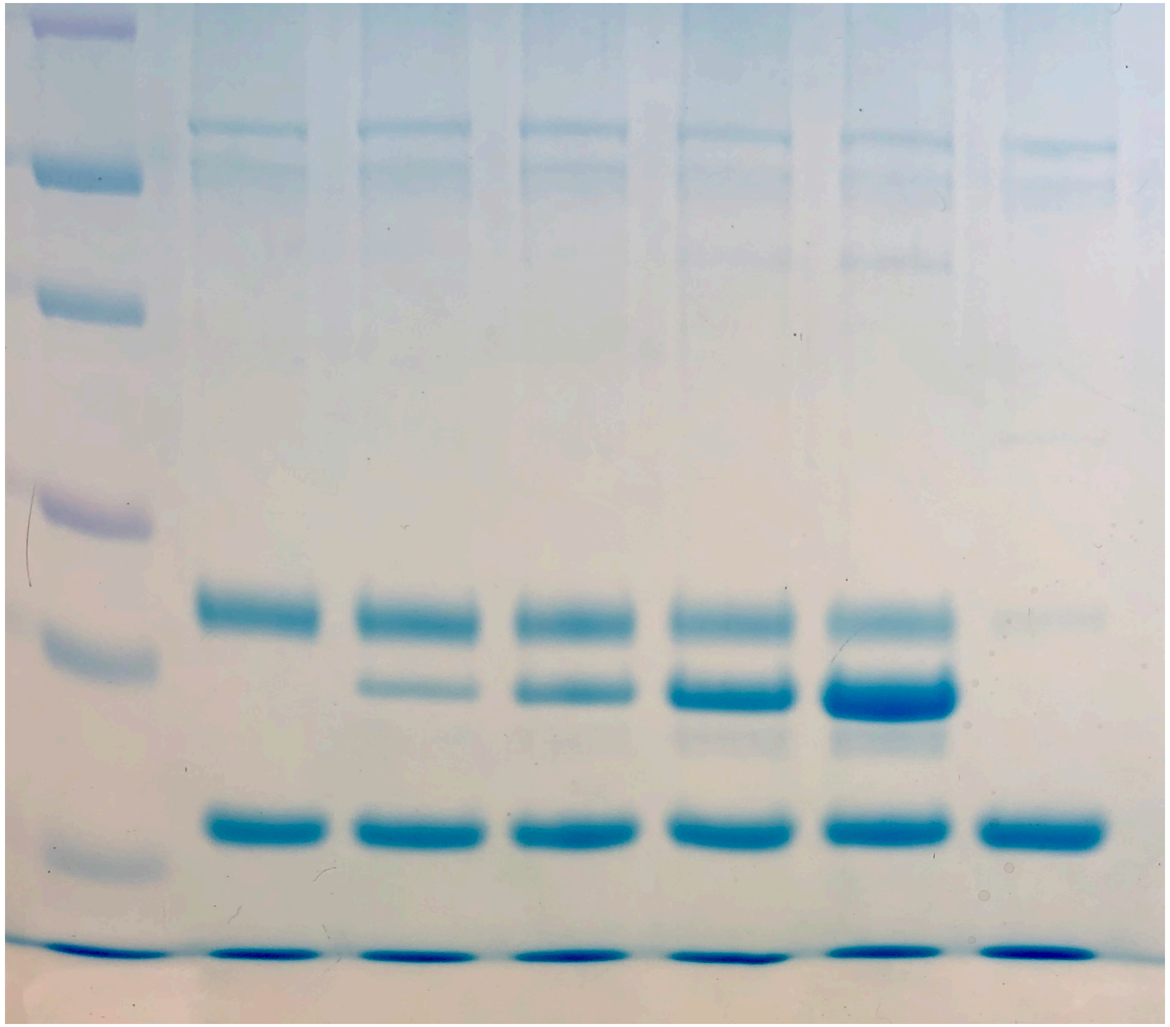

Supplement: Supplementary file 4 — Source Data [file 41467_2020_16211_MOESM4_ESM.zip › SourceDataFile_NCOMMS-19-35633A/OriginalGels-SourceDataFile.pdf]
