# Supplementary figures and images for: Legionella effector MavC targets the Ube2N~Ub conjugate for noncanonical ubiquitination
Source: Nat Commun. 2020 May 12;11:2365. doi: 10.1038/s41467-020-16211-x (PMC7217864; doi:10.1038/s41467-020-16211-x)

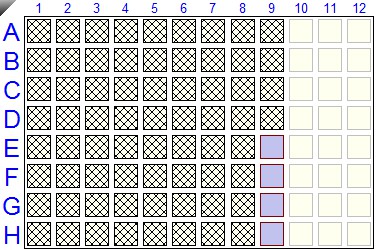

Supplement: Supplementary file 4 — Source Data [file 41467_2020_16211_MOESM4_ESM.zip › SourceDataFile_NCOMMS-19-35633A/BLI-SourceData/UbSSUbE2N with MavC FL/SensorPlate.jpg]

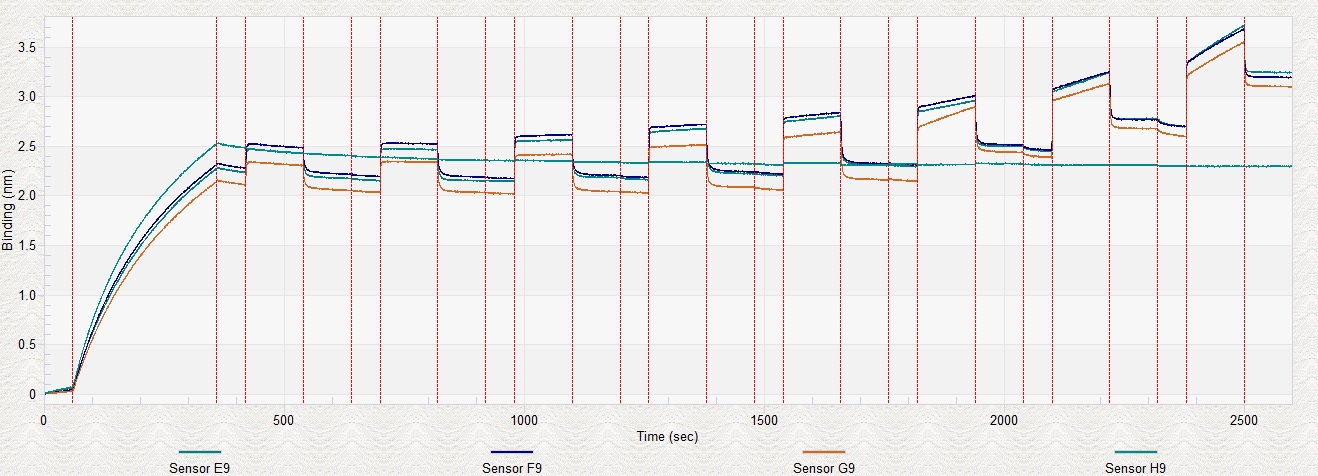

Supplement: Supplementary file 4 — Source Data [file 41467_2020_16211_MOESM4_ESM.zip › SourceDataFile_NCOMMS-19-35633A/BLI-SourceData/UbSSUbE2N with MavC FL/190807_Assay_1.jpg]

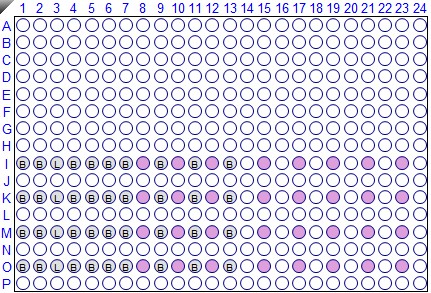

Supplement: Supplementary file 4 — Source Data [file 41467_2020_16211_MOESM4_ESM.zip › SourceDataFile_NCOMMS-19-35633A/BLI-SourceData/UbSSUbE2N with MavC FL/Plate1Definition.jpg]

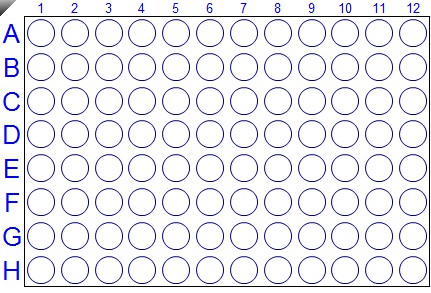

Supplement: Supplementary file 4 — Source Data [file 41467_2020_16211_MOESM4_ESM.zip › SourceDataFile_NCOMMS-19-35633A/BLI-SourceData/UbSSUbE2N with MavC FL/Plate2Definition.jpg]
